# Monster left circumflex artery fistula closure by vascular plug in a patient with post‐COVID‐19 myocardial infarction

**DOI:** 10.1002/ccr3.6579

**Published:** 2022-11-15

**Authors:** Peyman Izadpanah, Zahra Hooshanginezhad, Mohammad Shojaie

**Affiliations:** ^1^ Department of Cardiology Shiraz University of Medical Sciences Shiraz Iran; ^2^ Cardiology Department Jahrom University of Medical Sciences Jahrom Fars Iran

**Keywords:** coronary artery fistula, endovascular closure, vascular plug

## Abstract

Herein, we report a man with a huge coronary fistula connecting the left circumflex coronary artery to the right ventricle. During the follow‐up, the patient developed progressive symptoms of heart failure nonresponsive to medical treatment. Therefore, an endovascular closure with a vascular plug was successfully done for him. Transcatheter vascular plug occlusion can be considered as an alternative for closure of symptomatic high‐flow large coronary artery fistulas in patients with a high risk of surgery and chance of coil dislocation, embolization, or unavailability of proper coils.

## Graphical abstract

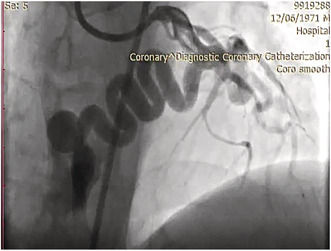

## 1 INTRODUCTION

Coronary arterial fistulas (CAFs) are abnormal connections between coronary arteries and great vessels or cardiac chambers; however, CAFs involving the left side chambers are rare. It is usually congenital but can be iatrogenic or acquired in rare cases.^1^ The diagnosis is primarily incidental, with an incidence of 0.05%–0.25% in those who have undergone coronary angiography.^2^ The prevalence of CAFs in coronary arteries is as follows: Right coronary artery (RCA) is the most prevalent (50%) followed by left anterior descending (LAD) artery (35%–40%) and left circumflex artery (LCX) (5%–20%).^3^ Small CAFs are usually asymptomatic and have an excellent prognosis, while the large ones might become symptomatic and complicated. The most common symptoms of these conditions are dyspnea, palpitation, and chest pain.^4^ The clinical importance of coronary fistula mainly owes to volume overload leading to heart failure and steal syndrome, which in turn cause coronary ischemia without stenosis. It can also increase the possibility of thrombosis and embolization, leading to myocardial infarction and arrhythmia and an increased risk of endocarditis.^5^, ^6^ The presence of symptomatic large coronary fistula indicates surgical or catheter‐based closure. If patients are selected meticulously, the transcatheter approach is safe and acceptable.^7^ In this case, we successfully used a Hyperion vascular plug to close a giant LCX fistula draining into the right ventricle.

## 2 CASE REPORT

**FIGURE 1:**
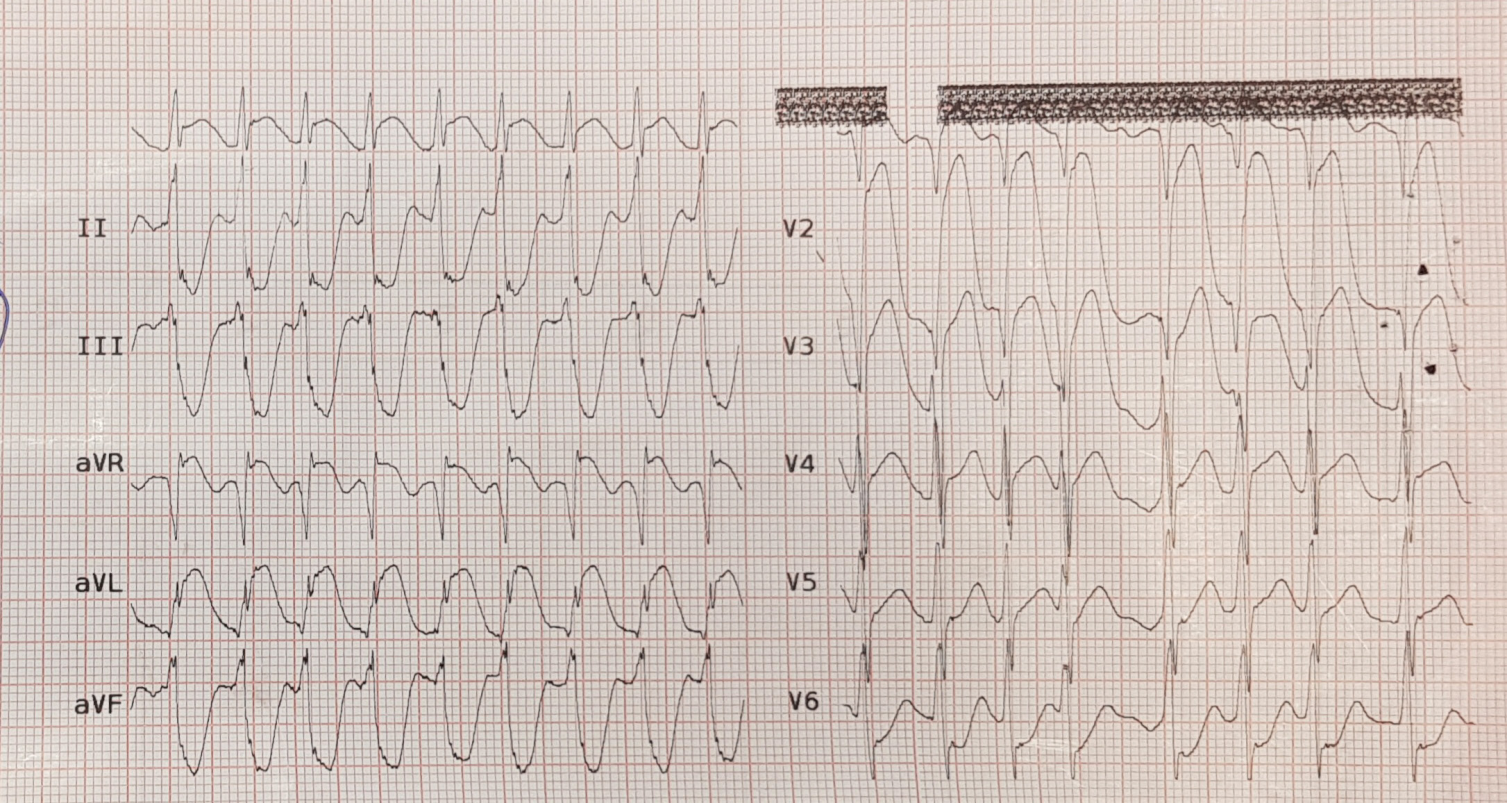
Electrocardiogram showing RBBB and anterior STEMI

This report represents a 50‐year‐old man referred to the emergency room of Peymaniyeh Hospital, Jahrom, Iran, with typical compressing chest pain radiating to his left shoulder accompanied by cold sweating. On admission, vital signs were as follows: blood pressure 95/70, heart rate: 130, and respiratory rate: 24. Electrocardiography was done upon arrival, revealing right bundle branch block (RBBB) and ST‐segment elevation in anterior leads (anterior STEMI, Figure 1).

**FIGURE 2:**
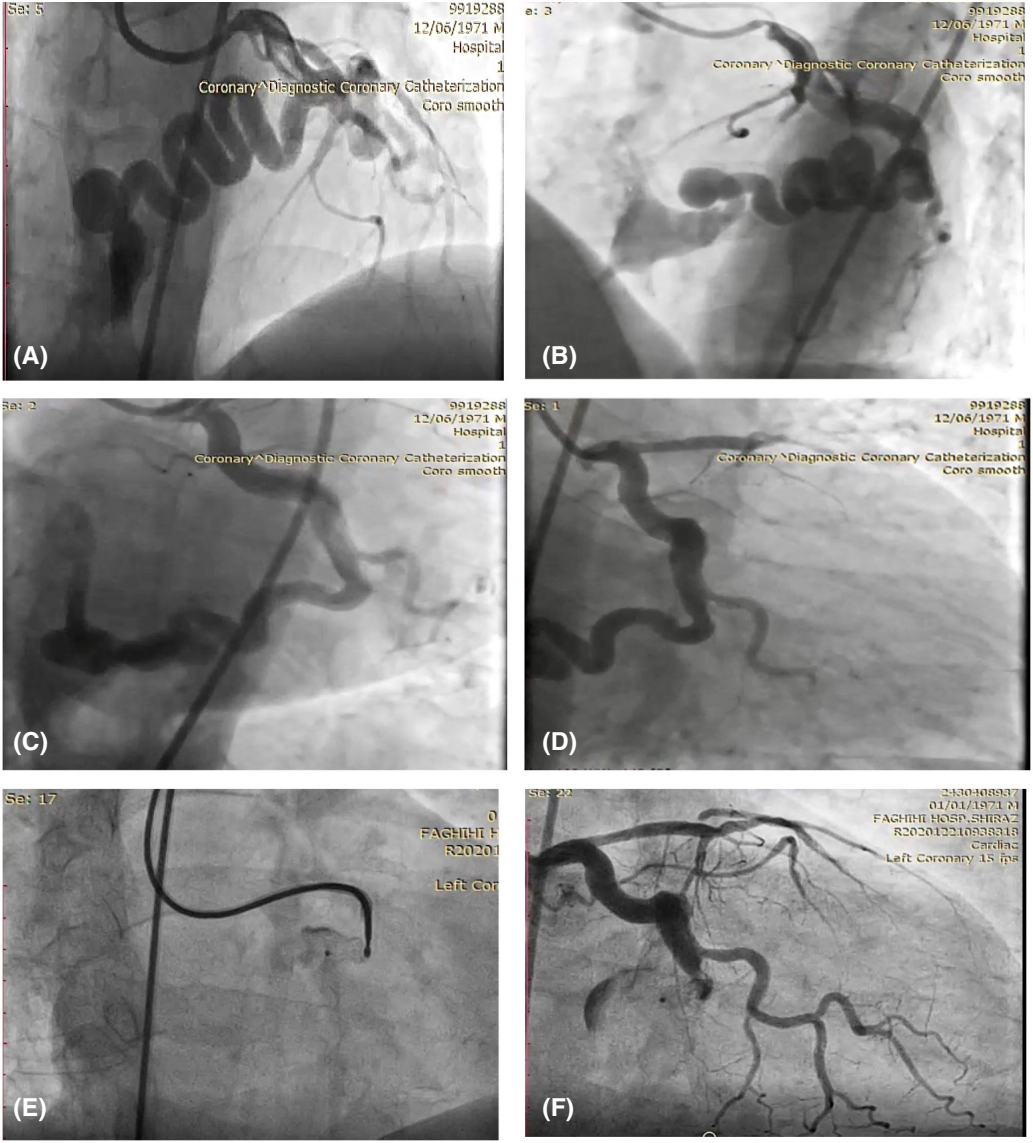
Angiographic findings of Monster LCX fistula: (A) AP cranial view; (B) LAO cranial view; (C) LAO caudal view; (D) RAO caudal view; (E) Entering the proximal part of the fistula; (F) Successful closure of the fistula with the Hyperion vascular plug

Transthoracic echocardiography showed anterior and apical wall hypokinesia with a left ventricular ejection fraction (LVEF) of about 30%. In less than an hour, the patient was sent to the catheterization laboratory for emergency coronary angioplasty, which showed total occlusion in the middle part of the LAD. Moreover, a giant CAF, about 6–8 mm, connecting the LCX to the right ventricle was detected (Figures 2A–D). Primary percutaneous intervention (PPCI) was successfully performed on the LAD, as the culpable vessel. LAD stenting was done with an Orsiro drug‐eluting 2.5 × 26 mm stent (Biotronik). During the PPCI, pulse oximetry revealed hypoxemia with an O_2_ saturation of about 81%. In the workup for the etiology of this hypoxemia, the polymerase chain reaction (PCR) result for the SARS‐CoV‐2 virus was positive. The chest computed tomography (CT) scan later showed bilateral mild pulmonary involvement, and the patient was isolated for observation for 1 week, during which his condition was stable. Successful post‐dilation of the LAD stent was done with Sapphire II NC 2.75 × 10 mm percutaneous transluminal coronary angioplasty (PTCA) balloon (OrbusNeich) 2 weeks later, resulting in a stable condition. In the follow‐up, the patient complained of progressive dyspnea on exertion with functional New York Heart Association (NYHA) class II/IV despite the optimal medical management of heart failure. Transthoracic echocardiographic evaluation was done after 12 weeks. The following data were obtained: left ventricle (LV) was dilated with moderate LV systolic dysfunction (LVEF: 35%); the right atrium and right ventricle (RV) were dilated, accompanied by normal RV function. Mild mitral and tricuspid valve regurgitation was noted. Systolic pulmonary arterial pressure (SPAP) was found to be 40 mmHg, and a significant left to right shunt with Qp/Qs: 2.1 was detected caused by the giant coronary artery to RV fistula. The cardiovascular team consisting of cardiac surgeons and interventional cardiologists discussed the case. Finally, an interventional transcatheter closure was decided as the next step, considering the patient's condition.

At the interventional closure session, an attempt was initially made to occlude the fistula by a detachable coil (EV3 FC‐8‐15‐3D Axium Prime Detachable Coil), but because of the high flow in the LCX fistula and the risk of embolization to the right heart, the release of the coil was not possible. Considering the unavailability of other types and sizes of coils, the patient was then scheduled for fistula closure by a vascular plug. Among the available vascular plugs, the Hyperion vascular plug with Hub (C‐PL 06, waist diameter: 6 mm, length of waist: 6.5 mm, compatible with the Orbit‐V delivery system 6F and larger) was chosen. The femoral artery approach was adopted via the guiding catheter Left Judkins 7 f (LJ 7F); however, despite multiple attempts with different angioplasty guidewires, the passage of the wire through the fistula was unsuccessful because of severe tortuosity and various loops in the fistulous tract. Afterward, a hydrophilic wire (0.035′ × 260 CM Angled Terumo Radifocus Guide Wire) was used to cross the fistula completely. Nonetheless, this approach failed as well. At this point, the femoral arterial sheath was changed to a long sheath (75 cm 7F), and the mentioned hydrophilic guidewire (0.035′ × 260 CM) was then passed through and advanced as far as possible by the support of a Guidion Hydro‐Biotronk extension catheter (G70F25150). Subsequently, the Guidion extension catheter was removed, and the vascular plug delivery system was placed in the LCX near the fistula. Unfortunately, the delivery system was relatively short (1–2 cm), and the device could not be released at the proper site. Thereupon, delivery of the vascular plug was planned to take place via a right Judkins guiding catheter 7F (RJ 7F). At this point still, the main issue was to maintaining the hydrophilic guidewire in the LCX fistula while changing the guiding catheter. To resolve this problem, the Guidion extension catheter was again inserted and pushed into the fistula, and then, the hub and its distal part were caught. Then, the guiding catheter LJ 7F was carefully removed and replaced by RJ 7F under fluoroscopy guidance and advanced on the Guidion‐guidewire complex. Then, while the distal end of the equipment was utterly fixed, the guiding wire was pushed gently into the LCX artery with subtle clockwise rotations until the tip entered the proximal part of the fistula (Figure 2E). The Guidion extension catheter was then removed, and the vascular plug was loaded. After careful evaluation for accurate positioning of the vascular plug, the guiding catheter was slightly pulled back. In this step, a localized dissection occurred in the proximal part of the fistula, and the patient complained of mild chest pain. The vascular plug was released with the slow withdrawal of the guiding catheter and opened in the proximal portion of the fistula just distal to the large Optus marginal branch (OM1). After the device was released, a mushroom‐shaped vascular plug was shown distal to OM1 without ante‐grade flow in the fistula in multiple angiographic views (Figure 2F). Also, a small localized no flow impediment dissection (type A) was evident in the ostio‐proximal part of the OM1. The patient was stable and free of chest pain after the procedure. In the 6‐month follow‐up, the patient was asymptomatic, and LVEF was improved to 45%.

## 3 DISCUSSION

Coronary arterial fistulas are abnormal communications between coronary arteries and are mainly congenital. Coronary blood flow is usually shunted via this unusual pathway into a cardiac chamber or great vessel.^8^, ^9^ Treatment is indicated in the presence of LV dysfunction or myocardial ischemia. Therapeutic modalities include percutaneous treatment and surgery. Surgical interventions have shown promising results. The catheter‐based technique is an alternative to the surgical approach. If case selection is performed judiciously, percutaneous closure has a desirable immediate and long‐term outcome comparable to the surgical method.^10^

Commonly, two types of materials are used for transcatheter closure of CAFs, including materials for embolization and occlude devices. Embolization materials include gel foam, coils, polyvinyl alcohol, and glue microspheres. Among these, coils are commonly used in CAF closure. Coils are available in various sizes, lengths, and thicknesses and can be easily delivered; however, they have embolization risk. Despite the unintended embolic incidence, which made these agents unsafe to use in some problematic, high‐flow fistulas, they remained the best choice for interventional CAF closure in most cases. On the contrary, occluder devices require relatively large sheaths for delivery since they are bulkier. These characteristics make occluders more challenging for use in tortuous and lower diameter structures. However, they can be released in a controlled fashion and remain a good option considering the lower rate of embolization.

Vascular plugs are self‐expanding, easy‐to‐use devices. They have the features of both embolic materials and occluder devices. Vascular plugs were previously used successfully for the closure of high‐flow extra cardiac vessel communications. Compared to coils, vascular plugs are preferred more in large extracardiac lesions.^11^ They have been used off‐label for the closure of large intra‐cardiac vascular connections. They have a lower dislocation risk than coils; however, the risk of vessel rupture is higher because of the need for a larger sheath to insert. Despite all these, they remain a good option in some cases where we cannot insert a coil successfully (because of the unavailability or high risk of embolization). In our case, a huge LCX to RV fistula, we successfully used a Hyperion vascular plug after an unsuccessful attempt to insert the coil.

## 4 CONCLUSION

Most cases of CAFs are asymptomatic and need no intervention; however, the large and symptomatic CAFs need either surgical or interventional closure. Coils are the most used devices for CAF closure. However, transcatheter occlusion by vascular plugs, which are mostly used for closure of high‐flow extracardiac vascular fistulas, is an alternative method that can be considered in cases with high surgical risk as well as a high chance of coil dislocation and embolization or unavailability of appropriate coils for transcatheter closure. To be able to compare the positive and negative aspects of using these methods in large CAFs and to determine the superiority of one over the other, more studies are needed.

## AUTHOR CONTRIBUTIONS

Peyman Izadpanah involved in methodology, validation, resources. Zahra Hooshanginezhad involved in validation, investigation, resources, writing case report, review, and editing. Mohammad Shojaie involved in methodology, validation, investigation, resources, writing case report, review, editing, and supervision. All authors have read and agreed to the published version of the manuscript.

## CONFLICT OF INTEREST

The authors declare no conflict of interest.

## CONSENT

Written informed consent was obtained from the patient to publish this report in accordance with the journal's patient consent policy.

## Data Availability

The data presented in this study are available upon request from the corresponding author.
